# Generic Workflow of a Highly Effective and Easy Anther Culture Method for Both Japonica and Indica Rice

**DOI:** 10.3390/plants13172531

**Published:** 2024-09-09

**Authors:** Guimei Guo, Shisen Liu, Shuwei Zhang, Linian Yang, Yingjie Zong, Nigel G. Halford, Ting He, Runhong Gao, Zhenzhu Guo, Longhua Zhou, Chenghong Liu, Shujun Wu, Zhiwei Chen

**Affiliations:** 1 Shanghai Key Laboratory of Agricultural Genetics and Breeding (21DZ2271900), Key Laboratory for Safety Assessment (Environment) of Agricultural Genetically Modified Organisms of Ministry of Agriculture and Rural Affairs (Shanghai), Biotechnology Research Institute of Shanghai Academy of Agricultural Sciences, Shanghai 201106, China; guoguimei@saas.sh.cn (G.G.); l406773972@outlook.com (S.L.); zhangshuwei@saas.sh.cn (S.Z.); lnyang1314@163.com (L.Y.); zongyingjie@saas.sh.cn (Y.Z.); heting@saas.sh.cn (T.H.); gaorunhong@saas.sh.cn (R.G.); gzzsk173@163.com (Z.G.); zhoulonghua@saas.sh.cn (L.Z.); liuchenghong@saas.sh.cn (C.L.); 2 Rothamsted Research, Harpenden AL5 2JQ, UK; nigel.halford@rothamsted.ac.uk; 3 Crop Breeding & Cultivation Research Institute, Shanghai Academy of Agricultural Sciences, Shanghai 201403, China; wushujun@saas.sh.cn

**Keywords:** *Oryza sativa* L., microspore, callus induction, plantlet regeneration, ploidy

## Abstract

As one of the most important staple crops in the world, rice plays a pivotal role in world food security. The creation of doubled haploids based on anther culture is an important technology for rice breeding. However, at present, rice anther culture technology still faces many problems, such as genotype dependency, especially genotypes of indica rice. In this study, fifteen rice genotypes, including twelve japonica rice genotypes and three indica rice genotypes, were randomly selected and used to study anther culture by using a modified M8 medium. The results showed that the total callus induction rates of these different rice genotypes ranged from 0.81 to 13.95%, with an average of 6.64%, while the callus induction rates calculated for the top ten highest callus inductions for each rice genotype ranged from 2.75 to 17.00%, with an average of 10.56%. There were varying gaps between the total callus induction rates and the callus induction rates in these different rice genotypes. The fact that the gaps for some rice genotypes were relatively large indicated that standard tiller or anther collection was not applicable to all rice genotypes and that there was still a lot of room for improvement in the callus induction rate of some rice genotypes through optimization of the sampling method. The plantlet regeneration rates ranged from 12.55 to 456.54%, with an average of 200.10%. Although there were many albinos from anther culture for some rice genotypes, these would still meet the requirement if the rice genotypes had higher callus induction rates or regeneration rates. The percentages of seed setting of regenerated green seedlings ranged from 14% to 84%, with an average of 48.73%. Genetic diversity analysis showed that the genetic background of these different rice genotypes was representative, and the phylogenetic tree and Principal Component Analysis (PCA) divided them into indica and japonica types. Therefore, in this study, an anther culture method suitable for both indica and japonica rice genotypes was established, which could improve doubled haploid breeding in rice.

## 1. Introduction

It is well known that homozygous recombinants with stable traits can be obtained in one year using haploid technology, greatly shortening the breeding cycle, and making the technology very popular among breeders [1,2]. In addition, haploid technology has important applications in research on crop genetics and breeding, including the creation of new and homozygous germplasm in combination with distant hybridization, the construction of permanent populations for genetic map-based gene cloning, their use as receptors for transgenesis and gene editing systems, the rapid identification of recessive mutant genes, mutagenesis and directional improvement, epigenetics, and so on. Therefore, since the 1960s, the technology for producing haploids has been developed in various plants or crops. So far, haploid technology has been established in more than 200 species of *Poaceae*, *Solanaceae*, and *Cruciferae*, and about 300 doubled haploid (DH) crop varieties have been bred based on this technology [2,3].

Rice is one of the world’s most important food crops and a staple food in Asian countries, making it essential for global food security because it supports more than half of the world’s population [4]. The use of haploid technology in rice, based on anther culture, was reported as early as 1968 [5]. Subsequently, haploid technology of rice based on microspore culture was reported by a Chinese scientist [6]. These technologies have played an important role in the creation of haploids and the breeding of new DH varieties in rice [2]. Although there are good examples of breeding DH varieties in both japonica and indica rice, it is still generally considered that haploid induction in indica rice is more difficult than in japonica rice [2]. It is also difficult to evaluate relevant phenotypic traits for in vitro haploid induction in rice because of empirical uncertainties and cultural limitations, of which there are many, so that the molecular mechanisms of in vitro haploid induction have been difficult to reveal. In addition, due to the rapid development of molecular biology, many new technologies have received more attention, such as molecular marker-assisted breeding, transgenic techniques, genome-wide selection, gene editing, etc. It was not until the breakthrough of the molecular mechanism of in vivo haploid induction and its verification in other crops that haploid induction technology once again received great attention [7,8]. Although induction lines for in vivo haploid induction had also been successfully created in rice [9], this also inspired researchers to refocus on in vitro haploid induction techniques based on anther/microspore culture.

There are many factors that affect anther culture, including genotype, microspore development stage, pretreatment, basic medium, hormones, and other external additives [2,10]. There are also many steps involved in the whole process of anther culture, so it is difficult to make direct comparisons between different culture experiments. Previously, we established an anther culture method based on N6 medium for japonica rice [11]. In this study, we aimed to establish an anther culture method suitable for both indica and japonica rice. This would enable us to more accurately assess the differences in their responses to anther culture and provide a basis for optimizing rice varieties with poor anther culture ability and elucidating their molecular mechanisms.

## 2. Results

### 2.1. Different Responses in Callus Induction for Different Rice Genotypes

Due to the factors of different tiller collection times for different rice genotypes, different amounts of suitable tillers, contamination, etc., it was difficult to keep the number of inoculated anthers and inoculation time consistent, especially when there were a large number of rice genotypes. Therefore, when comparing the anther culture of different rice genotypes, it could only be guaranteed that the cultivation of each rice genotype could be completed under the same procedure and culture conditions, while it might not be possible to ensure that all rice genotypes were completed simultaneously. Callus induction in anther culture in this experiment is summarized in Table 1, and the experimental manipulation was relatively good because there were no obvious contaminations. The total callus induction rates ranged from 0.81 to 13.95%, with an average of 6.64%, and this indicated that there were large differences in callus induction among different rice genotypes. Looking at the three rice genotypes with the lowest total callus induction rates, including Q3, Q4, and Q7, their situations were different. For example, the proportion of triangular flasks without induced calli in Q4 and Q7 was significantly higher, while it was lower in Q3. This suggested that, in addition to the rice genotype, there might have been problems with the timing of the tiller collection or the evaluation of the microspore development. In order to further analyze the differences in callus induction among different rice genotypes, the data of the ten triangular flasks with the highest callus induction rates for each rice genotype were selected for statistical analysis.

**Figure 1:**
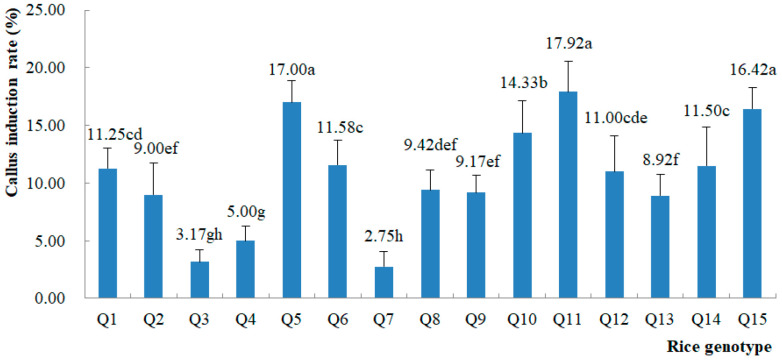
Callus induction rate of different rice genotypes. Different letters mean significant differences in callus induction rate between different rice genotypes.

The one-way ANOVA showed that there were significant differences in callus induction rates between the different rice genotypes (Table S1), and the comparative analysis also showed their exact differences (Figure 1). This indicated that the responses to callus induction differed between the genotypes. Q5, Q11, and Q15 had the highest callus induction rates, with no significant differences between them, while Q3, Q4, and Q7 had the lowest callus induction rates. Q3 and Q7 were both indica rice varieties. At the same time, the callus induction rate of the indica rice genotype of Q6 was relatively high, reaching 11.58%, while the japonica rice, Q4, had a lower callus induction rate of 5.00%. This indicated that there was no obvious selectivity for indica or japonica rice by using this callus-inducing medium and culture program and that the main effects might be related to individual genotypes and their microspore development stage. Comparing the callus induction rates between the total inoculated anthers and the inoculated anthers in the ten triangular flasks with the highest callus induction rates for each rice genotype, some of them were relatively similar, while others differed greatly. It was inferred that the standard of evaluation of the microspore development stage for anther inoculation might not be applicable to all rice genotypes or might not be at the most suitable time for some of them.

### 2.2. Different Responses in Plantlet Regeneration for Different Rice Genotypes

The plantlets regenerated from the induced calli for each rice genotype after two weeks of differentiation are summarized in Table 2. Their green plantlet regeneration rates ranged from 8.96% to 407.59% with an average of 140.47%, which indicated that there were large differences in green plantlet regeneration between the genotypes. Meanwhile, there was also a problem of the presence of albino plantlets, but this was also genotype-dependent, with albino plantlet regeneration rates ranging from 3.42% to 113.17%, and the ratio of green to albino plantlets ranging from 0.16 to 8.33. Although these albino plantlets would be discarded, they were included in the calculation of the plantlet regeneration. In addition, there were a great number of plantlets with green spots for some rice genotypes. These were also included in the calculation of the differentiation ability. The Pearson correlation coefficients showed that there were no significant correlations between any two of the three traits of total callus introduction rate, green plantlet regeneration rate, and albino plantlet regeneration rate, although the total callus induction rate was negatively correlated with both of the other two traits, and the green plantlet regeneration rate and albino plantlet regeneration rate were positively correlated (Table S2). This further indicated that callus induction and plantlet regeneration were both genotype-dependent, but that these two processes were independent from each other.

### 2.3. Ploidy Identification of Regenerated Plants

**Figure 2:**
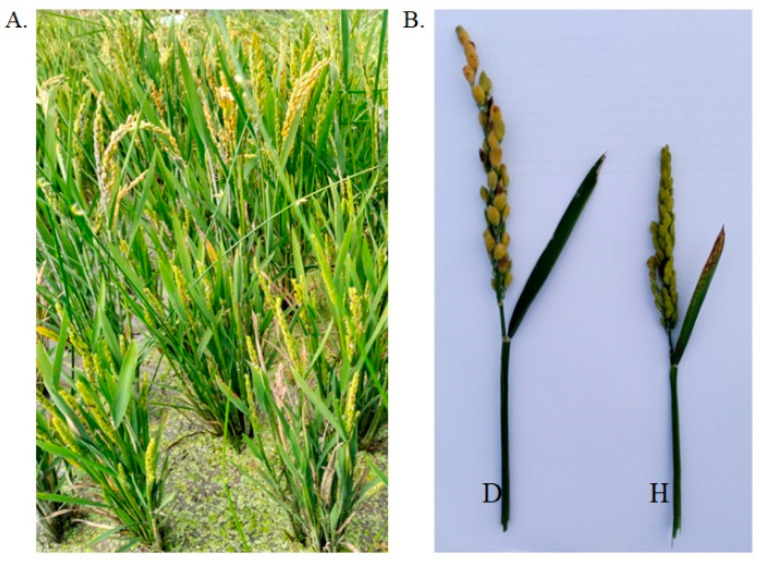
Ploidy identification of regenerated plants in the field at grain-filling stage. (**A**) Their growth in fields. (**B**) The comparison of diploid and haploid rice panicles. “D” means diploid and “H” means haploid.

Flow cytometry and guard cell length methods are usually used in the large-scale ploidy identification of regenerated plants derived by anther and microspore cultures. However, ploidy identification here was mainly judged according to phenotype in the field. Although it was attempted to judge the ploidy according to leaf size and plant height at the seedling stage, it was very difficult to discriminate according to these traits in the field. In contrast, plant height, spikelet size, and seed setting could be well distinguished at the grain-filling stage, so in addition to the use of seed setting as the identification of ploidy, the other two traits were also used as important references (Figure 2) [12]. In addition to Q3, 50 regenerated plants were randomly selected for each rice genotype and used for ploidy identification. Considering the very few tetraploid plants from anther culture, they were not listed separately here, but were included in the diploid count. The ploidy levels of regenerated plants of the fifteen rice genotypes are shown in Table 3; the percentages of diploid plants ranged from 14% to 84%, with an average of 48.73%. This suggested that the ploidy of regenerated plants might also be genotype-dependent. In addition, it was found that the regenerated plants could survive after being transplanted directly into fields from tissue culture conditions, and in this experiment, the survival rates of plants of the fifteen rice genotypes were almost all 100%.

### 2.4. SNP Markers and Genetic Diversity Analysis

A total of 49,676 SNPs were obtained, and all of them belonged to the bi-allelic type. The effective number of alleles (Ne) ranged from 1.0689 to 2, with an average of 1.3892, and there were 957 SNPs with a Ne value greater than or equal to 1.9882, and even 187 SNPs with a Ne value equal to 2. The major allele frequency (MAF) ranged from 0.0333 to 0.5, with an average of 0.1687. The Hardy–Weinberg equilibrium *P*-value (HW-P) ranged from 4.43 × 10^−5^ to 1, with an average of 9.73 × 10^−2^, and there were 44,796 SNPs with a HW-*P* value of less than 0.05. The polymorphism information content (PIC) ranged from 0.0624 to 0.3750, with an average of 0.2215, and there were 25,170 SNPs with a PIC value of less than 0.25, while others had a PIC value of between 0.25 and 0.38 (about 49.33%). The expected heterozygosity (He) ranged from 0.0644 to 0.5, with an average of 0.2623, and the observed heterozygosity (Ho) ranged from 0 to 1, with an average of 0.0137. There were 44,153 SNPs (about 88.88%) with an Ho value of zero and two SNPs with a Ho value of 1. The nucleotide diversity (π) ranged from 0.0677 to 0.5217, with an average of 0.2718. The above data suggested that the degrees of homozygosity of these rice varieties were already very high and that they had rich genetic backgrounds. Thus, the culture responses were also representative in the rice.

**Figure 3:**
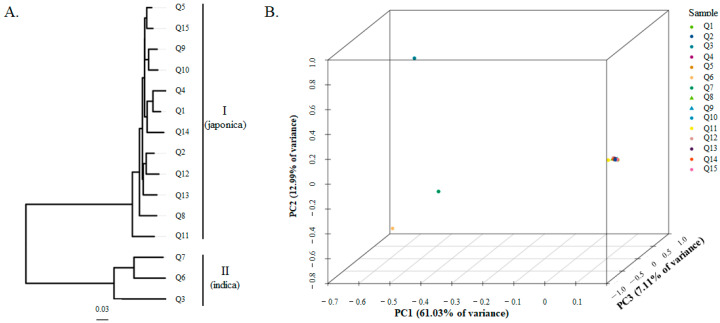
Phylogenetic tree and principal component analysis (PCA) of fifteen rice genotypes based on 49,676 SNP markers. (**A**) The phylogenetic tree constructed using the neighbor-joining method; (**B**) The PCA plot.

The phylogenetic tree showed that these rice varieties were clustered into two groups, named I and II (Figure 3A). This classification was just right to separate the indica and japonica rice varieties. The PCA showed that the first two principal components explained 81.13% of the variance, and they were 61.03% for PC1, 12.99% for PC2, and 7.11% for PC3 (Figure 3B). The two subspecies of indica and japonica rice could be separated by PC1.

## 3. Discussion

For a long time, genotype was considered to be the most decisive factor in anther culture of rice, and japonica rice had a higher regeneration efficiency than indica rice [2,10,13,14]. As a result, many researchers began to create induction lines through gene editing to solve the creation of DH plants for rice, especially indica rice [9,15]. While the genotypic dependency of anther culture could be broken by optimization of the culture medium, according to our experience in anther or microspore culture of barley [16], we preferred the idea that different rice genotypes might have different preferences for culture media and that genotypic dependency could be solved by providing suitable culture media.

In this study, a modified M8 medium was used for callus induction, and the results showed that callus could be induced in both indica and japonica rice genotypes, with no obvious differences between the two subspecies, although only three indica rice genotypes were included. Of course, the callus induction rates were different for the different genotypes. Therefore, we inferred that bias against the anther culture of indica rice might be due to the use of a medium preferred for japonica rice. There have also been reports about the good effects of M8 and its derived media on anther culture in both indica and japonica rice genotypes [17,18], so it might be more objective to use the modified medium based on the M8 basic medium to evaluate the two subspecies of indica and japonica rice. In addition, for the genotypic dependency in callus induction, we also inferred that the uniform standard of sampling and inoculation could not obtain the microspores at the optimal stage in anther culture for all rice genotypes and that this would also lead to different callus induction rates in different genotypes. In fact, there have been different reports on the optimal microspore development stage for anther culture in rice, such as early- to mid-uninucleate [12,19] or late-uninucleate to early-binucleate [20,21]. Therefore, it might be necessary to adjust or find the optimal microspore development stage for each rice genotype separately to alleviate the genotypic dependency in callus induction.

It is well known that not all calli can be differentiated into plantlets but only the embryonic calli amongst them. Thus, it was hard to establish a relationship between callus induction and plantlet regeneration because it was impossible to confirm whether the calli were embryogenic. In fact, in this study, there was even a negative correlation between callus induction and green plantlet regeneration, although it was not statistically significant. He et al. [22] also found that there was no significant correlation between callus induction frequency and plantlet differentiation frequencies (both of albino and green plantlets), and the QTLs controlling callus induction frequency and plantlet differentiation frequencies were independent. All of the above showed that callus induction and plantlet regeneration were two independent processes under the current conditions of anther culture. In addition, we also found that albino plantlets were an unavoidable problem in the process of plantlet regeneration, not only because the albino plantlet regeneration rate was high, but also because the albino plantlet regeneration rates of some rice genotypes, such as Q3, Q6, Q8, and Q10, exceeded their green plantlet regeneration rates. This problem has also been described by other researchers [2,14]. Meanwhile, a clear example of a problem highlighted by this study was the result for the Q6 genotype, where the calli were not transferred in time, resulting in all regenerated plantlets being albinos. Consistent with this, it has been already been reported that shortening the culture time can reduce the incidence of albinos [23]. Of course, in practice, the production of some albino plantlets would not be a problem if higher callus induction and green plantlet regeneration rates could be ensured.

It is worth mentioning that indoor seedling refining was previously usually carried out by using hydroponic culture before transplanting into fields, but we found that the regenerated rice seedlings could be transplanted directly into fields with a survival rate of nearly 100%. In addition, in order to simplify the anther culture process, artificial chromosome doubling was not conducted in this study, but it was found that spontaneous chromosome doubling occurred in all rice genotypes, but there were large differences in rates between the genotypes. There were seven rice genotypes with a diploid percentage of more than 50%, six between 30 and 50%, and only two were below 20%. There have been differing reports on spontaneous chromosome doubling of rice in anther culture, some giving the percentage of diploid plants at 20–30% [24], others at 50–60% [25], while some even give it at 90–99% [26]. We inferred that spontaneous chromosome doubling of rice in anther culture under the same conditions was related to the rice genotypes, but that the method of ploidy identification used was also important.

## 4. Materials and Methods

### 4.1. Plant Materials

A total of fifteen rice genotypes were used in this study, of which eleven were from the National Mid-term Bank for Rice (Fuyang of Zhejiang Province, China), and the remaining four were from the Crop Breeding and Cultivation Research Institute of Shanghai Academy of Agricultural Sciences (Shanghai, China) (Table 4). Among the first eleven rice genotypes, three model rice varieties were included, namely Nipponbare, 93–11, and Zhonghua 11, while the rest were excellent cultivars with relatively large promotion areas in the Yangtze River Delta region of China. The last four rice genotypes were the core parents of the rice breeding team in the Institute. The seedlings of these rice genotypes were planted in the fields of Chonggu Experimental Base of Shanghai Academy of Agricultural Sciences (31.19° N, 121.20° E) in June 2023.

### 4.2. Tiller Collection and Cold Pretreatment

Healthy tillers at the booting stage were collected from each rice genotype in the morning between 8:00 and 10:00 a.m., ensuring that the development stage of microspores in the florets at the middle of the panicles was at the late-uninucleate to early binucleated stage. The collected tillers were trimmed neatly and wrapped in wet cheesecloth, then sealed in polyethylene bags to prevent desiccation. Then, they were incubated at 5 °C for one week for cold pretreatment.

### 4.3. Culture Media and Preparation

The callus induction medium (CIM) was based on M8 medium, with maltose, glutamine, hydrolyzed casein, 2,4-D, KT, NAA, and agar added to a final concentration of 50 g/L, 1 g/L, 1 g/L, 2.0 mg/L, 1.0 mg/L, 0.5 mg/L, and 6.0 g/L, respectively. The plantlet regeneration medium (PRM) was based on 1/2 MS medium, with maltose, KT, 6-BA, NAA, and agar added to a final concentration of 30 g/L, 2.0 mg/L, 1.0 mg/L, 0.5 mg/L, and 6.0 g/L, respectively. The rooting medium (RM) was also based on 1/2 MS medium, with sucrose, NAA, paclobutrazol, and agar added to a final concentration of 30 g/L, 0.4 mg/L, 3.0 mg/L, and 6.0 g/L, respectively. The pH of all media was adjusted to 5.8, and the media were then autoclaved at 121 °C, 0.11 Mpa for 20 min for further use.

### 4.4. In Vitro Anther Culture

Florets whose anther length reached one-third to one-half of the total floret length were selected. This corresponds roughly to the late-uninucleate to early binucleated stage of pollen development, which is the most sensitive stage for callus induction. The selected florets were disinfected with 10% sodium hypochlorite for 10 min and rinsed with sterile water 4~5 times. Then, the anthers within these florets were separated for inoculation, and 120 anthers were inoculated in each 50 mL triangular flask containing 20 mL CIM. The triangular flasks with the inoculated anthers were placed in an incubator at 26 °C and 60–75% relative humidity in the dark for callus induction, and the induced calli were counted after four weeks.

### 4.5. Plantlets Regeneration, Rooting and Ploidy Determination

The induced calli of each rice genotype were transferred to a 100 mL triangular flask containing 50 mL PRM, and the triangular flasks with the inoculated calli were then placed in a culture room for plantlet regeneration at 24 °C, 60–75% relative humidity, and for a 16 h photoperiod. Due to the variable size of the calli induced from different anthers, it was not possible to put calli from the same number of anthers into each triangular flask containing plantlet regeneration media (PRM). Therefore, it was difficult to count the exact number of plantlets that could be differentiated from calli induced by every anther, and only overall estimates could be provided. After two weeks, regenerated green plantlets more than 2 cm in height were transferred to a 200 mL jar containing 70 mL RM, and the jars with inoculated plantlets were placed in the same conditions of plantlet regeneration for rooting. The regenerated plantlets were counted when they were inoculated into the RM, the albino plantlets were only used for counting and then discarded, while the regenerated plantlets of Q6 were counted by using other anther-induced calli (seven weeks of callus induction). After six weeks, the intact and robust rice plants were removed, and the medium around the roots was washed off. About fifty plants of each rice genotype were randomly selected and sent to the Hainan Experimental Base (Lingshui of Hainan Province, China) (18.52° N, 110.02° E) for transplanting in December 2023. The ploidy of the regenerated plants was mainly identified according to seed setting at the filling stage, and the plant height and spikelet size were also used as references [12,24].

### 4.6. Genotyping-by-Sequencing (GBS)

The GBS analysis was performed mainly according to Chen et al. [27]. The total genomic DNA of each rice genotype was extracted, and the GBS analysis was then conducted by the Oebiotech Company (Shanghai, China) using the Illumina Nova platform (Illumina Inc., San Diego, CA, USA). The 150 bp pair-end raw reads were generated and uploaded to the NCBI database (PRJNA1118907). The clean reads of the samples ranged from 0.57 to 0.77-Gb, and their Q30 were all >93.90% (see Table S3). The local alignment of the clean reads of the fifteen rice genotypes to the reference genome of rice cv. Nipponbare (http://rice.uga.edu/index.shtml accessed on 26 January 2024) was conducted using BWA software (v0.7.17). SNP calling was performed using the HaplotypeCaller module of the Genome Analysis Tool Kit (GATK) software (v4.1.3), and only SNP markers with QualByDepth (QD) ≥10 were preserved [28]. The SNP markers were further filtered using vcftools (v0.1.16), and the SNPs with a sequencing depth <4, a minor allele frequency (MAF) <0.01, or a missing rate >20% were removed [29]. The filtered SNP markers were annotated using the SnpEff software (v4.1 g) [30].

### 4.7. Genetic Diversity Analysis

Genetic parameters for each of the filtered SNPs described above, including the Hardy–Weinberg equilibrium *P*-value (HW-P), observed heterozygosity (Ho), expected heterozygosity (He), polymorphic information content (PIC), nucleotide diversity (π), observed number of alleles (Na), and effective number of alleles (Ne), were calculated using vcftools (v0.1.16). The SNPs used in the phylogenetic tree analysis and principal component analysis (PCA) were filtered by requiring MAF < 0.05. The phylogenetic tree of fifteen rice genotypes was constructed using the neighbor-joining method [31]. The distance matrix was calculated using TreeBeST software (v1.9.2) [32], and the reliability of the tree was tested using the BootStrap method (repeated 1000 times) [33]. The PCA was performed using GCTA software (v1.26.0) [34].

## 5. Conclusions

**Figure 4:**
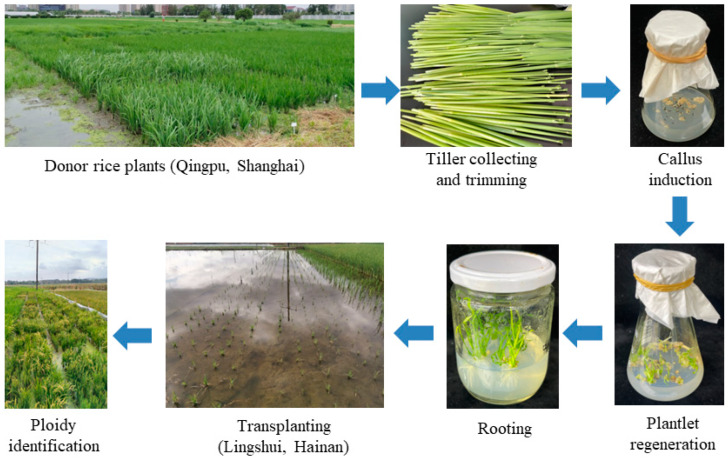
The workflow of anther culture in rice.

Here, we established an anther culture method suitable for different genotypes of both indica and japonica rice (Figure 4). This method used an optimized callus induction medium based on M8 basic medium, simplified the process of anther culture by collecting anthers according to anther length, and involved no artificial chromosome doubling or indoor seedling refining, so that anther culture could be easily scaled up for practical applications. The seed setting rate of thirteen of the fifteen rice genotypes was more than 30%, so spontaneous chromosome doubling could meet the needs of breeding in most cases. Of course, in the next step, it is necessary to optimize the sampling standard and callus induction period to further improve callus induction and green plantlet regeneration and expand the use of this genotype-independent anther culture method in rice.

## Figures and Tables

**Table 1:** Callus induction of different rice genotypes.

Code of Rice Genotype	Total Number of Triangular Flasks for Anther Inoculation	Total Number of Contaminated Triangular Flasks	Total Number of Triangular Flasks without Induced Calli	Total Number of Triangular Flasks with Induced Calli	Total Number of Anthers of Induced Calli	Total Callus Introduction Rate (%)
Q1	53	2	4	47	350	5.72
Q2	30	3	5	22	158	4.88
Q3	35	0	6	29	67	1.60
Q4	50	5	17	28	101	1.87
Q5	43	0	0	43	608	11.78
Q6	43	1	6	36	291	5.77
Q7	40	0	25	15	39	0.81
Q8	25	0	0	25	203	6.77
Q9	35	0	2	33	199	4.74
Q10	35	2	0	33	375	9.47
Q11	35	0	0	35	526	12.52
Q12	31	2	0	29	263	7.56
Q13	35	0	0	35	243	5.79
Q14	31	0	1	30	237	6.37
Q15	19	0	0	19	318	13.95

**Table 2:** Plantlet regeneration of different rice genotypes.

Code of Rice Genotype	Total Number of Anthers of Induced Calli	Number of Green Plantlets	Number of Albino Plantlets	Number of Green Spots	Green Plantlet Regeneration Rate (%)	Albino Plantlet Regeneration Rate (%)	Ratio of Green and Albino Plantlets
Q1	350	745	313	55	212.86	89.43	2.38
Q2	158	375	68	1122	237.34	43.04	5.51
Q3	67	6	37	8	8.96	55.22	0.16
Q4	101	77	60	ND	76.24	59.41	1.28
Q5	608	587	197	1033	96.55	32.40	2.98
Q6	132	24	93	ND	18.18	70.45	0.26
Q7	39	102	16	ND	261.54	41.03	6.38
Q8	203	57	135	29	28.08	66.50	0.42
Q9	199	226	156	85	113.57	78.39	1.45
Q10	375	355	389	946	94.67	103.73	0.91
Q11	526	48	18	113	9.13	3.42	2.67
Q12	263	136	78	ND	51.71	29.66	1.74
Q13	243	564	275	161	232.10	113.17	2.05
Q14	237	966	116	57	407.59	48.95	8.33
Q15	318	822	190	250	258.49	59.75	4.33

Note: “ND” means no data.

**Table 3:** Plantlet regeneration of different rice genotypes.

Code of Rice Genotype	Total Number of Transfer Plants	Number of Haploid Plants	Number of Diploid Plants	Percentage of Haploid Plants (%)	Percentage of Diploid Plants (%)	Ratio of Diploid and Haploid Plants
Q1	50	18	32	36	64	1.78
Q2	50	28	22	56	44	0.79
Q3	20	7	13	35	65	1.86
Q4	50	28	22	56	44	0.79
Q5	50	35	15	70	30	0.43
Q6	50	8	42	16	84	5.25
Q7	50	15	35	30	70	2.33
Q8	50	43	7	86	14	0.16
Q9	50	34	16	68	32	0.47
Q10	50	42	8	84	16	0.19
Q11	50	9	41	18	82	4.56
Q12	50	16	34	32	68	2.13
Q13	50	34	16	68	32	0.47
Q14	50	25	25	50	50	1.00
Q15	50	32	18	64	36	0.56

**Table 4:** Rice genotypes used in this study.

Code	Rice Genotype	Subspecies	Origin
Q1	Nipponbare	Japonica	National Mid-term Bank for Rice, China
Q2	Wuyunjing 7	Japonica	
Q3	93–11	Indica	
Q4	Zhonghua 11	Japonica	
Q5	Nanjing 46	Japonica	
Q6	Zhongzao 39	Indica	
Q7	Zhongjiazao 17	Indica	
Q8	Xiushui 134	Japonica	
Q9	Nanjing 9108	Japonica	
Q10	Nanjing 5055	Japonica	
Q11	Shangshida 19	Japonica	
Q12	7375	Japonica	Crop Breeding & Cultivation Research Institute of SAAS
Q13	Hudao 89	Japonica	
Q14	Huruan 1212kang	Japonica	
Q15	Huxiangruan 450	Japonica	

## Data Availability

Available at http://www.ncbi.nlm.nih.gov/bioproject/PRJNA1118907 (accessed on 1 June 2024).

## Supplementary Materials

The following supporting information can be downloaded at: https://www.mdpi.com/article/10.3390/plants13172531/s1, Table S1: One-way ANOVA of the callus induction rate; Table S2: Pearson correlations among callus induction, green, and albino plantlet regeneration rates; Table S3: Summary of DNA-seq data from seedlings of different rice genotypes.
