# Supplementary material for: Generic Workflow of a Highly Effective and Easy Anther Culture Method for Both Japonica and Indica Rice
Source: Plants (Basel). 2024 Sep 9;13(17):2531. doi: 10.3390/plants13172531 (PMC11397046; doi:10.3390/plants13172531)
Supplement: Supplementary file 1 [file plants-13-02531-s001.zip › plants-3142556-supplementary.docx]

Supplementary Materials

**Table S1.** One-way ANOVA of the callus induction rate.

|  | **Sums of Squares (SS)** | **Degrees of Freedom (df)** | **Mean Square (MS)** | ***F* value** | ***P*-value** |
| --- | --- | --- | --- | --- | --- |
| Between-group | 3016.451 | 14 | 215.461 | 46.003 | 0.000 |
| Within-group | 632.285 | 135 | 4.684 |  |  |
| Total | 3648.737 | 149 |  |  |  |

**Table S2.** Pearson correlations among callus induction, green and albino plantlet regeneration rates.

|  | **Green plantlet regeneration rate** | **Albino plantlet regeneration rate** |
| --- | --- | --- |
| Callus induction rate | -0.050 | -0.205 |
| Green plantlet regeneration rate |  | 0.155 |

**Table S3.** Summary of DNA-seq data from seedlings of different rice genotypes.

| **Code of rice genotype** | **Raw base (G)** | **Clean base (G)** | **GC content (%)** | **Q30 (%)** |
| --- | --- | --- | --- | --- |
| Q1 | 1.11 | 0.73 | 48.66 | 93.97 |
| Q2 | 1.03 | 0.70 | 49.23 | 94.15 |
| Q3 | 0.92 | 0.62 | 48.99 | 94.46 |
| Q4 | 1.05 | 0.70 | 49.83 | 94.26 |
| Q5 | 1.16 | 0.77 | 48.51 | 94.13 |
| Q6 | 0.98 | 0.68 | 48.76 | 94.17 |
| Q7 | 0.93 | 0.63 | 49.72 | 94.41 |
| Q8 | 1.11 | 0.74 | 49.70 | 94.37 |
| Q9 | 0.88 | 0.58 | 49.16 | 94.25 |
| Q10 | 0.87 | 0.60 | 48.66 | 94.07 |
| Q11 | 0.86 | 0.57 | 49.66 | 94.42 |
| Q12 | 0.92 | 0.61 | 48.94 | 93.77 |
| Q13 | 0.87 | 0.60 | 49.32 | 94.27 |
| Q14 | 1.01 | 0.64 | 54.72 | 94.64 |
| Q15 | 0.86 | 0.59 | 48.45 | 94.28 |
